# Instability of treated vertebrae after balloon kyphoplasty causing paraparesis in osteoporotic vertebral compression fracture: a report of two cases

**DOI:** 10.1007/s00586-012-2414-9

**Published:** 2012-07-03

**Authors:** Tetsuro Ohba, Shigeto Ebata, Devin Clinton, Kenske Koyama, Hirotaka Haro

**Affiliations:** 1 Department of Orthopaedic Surgery, University of Yamanashi, 1110 Shimokato, Chuo, Yamanashi, 409-3898 Japan; 2 Department of Orthopaedics, Vanderbilt University, Nashville, TN USA

**Keywords:** Osteoporotic vertebral compression fracture, Balloon kyphoplasty, Lower extremity paraparesis

## Abstract

**Purpose:**

To describe two unique cases of osteoporotic vertebral compression fracture (OVCF) treated with balloon kyphoplasty (BKP) that were complicated by spinal instability and resultant lower extremity paraparesis.

**Methods:**

Kyphoplasty was performed in two patients with OVCF that had persistent back pain despite a course of conservative care. Immediately following BKP, both patients had a marked improvement in back pain. However, they developed progressive bilateral lower extremity weakness. Lateral spine flexion–extension radiographs demonstrated instability, and polymethyl methacrylate did not adhere to the endplate of the treated vertebrae.

**Results:**

Both the patients underwent a hybrid fixation without a decompression. Postoperatively, both of them demonstrated gradual improvement in their neurological exam.

**Conclusions:**

To the best of our knowledge, this is the first report describing the development of spinal instability with resultant delayed paraplegia following BKP. This case report demonstrates another cause of neurological decline following BKP, in the absence of cement leakage.

## Introduction

Osteoporotic vertebral compression fracture (OVCF) is one of the most common complication of osteoporosis. Vertebral compression fractures affect an estimated 1.4 million people worldwide annually [1]. The burden of OVCFs can be substantial, resulting in chronic pain, marked reduction in health-related quality of life, and high health-care costs [1]. Over the past two decades, vertebroplasty has been developed to stabilize OVCF without the increased morbidity and mortality associated with open surgery [2]. Kyphoplasty, a modification of vertebroplasty, has theoretical advantages including focal kyphosis correction and lower complication rate [3]. Boonen et al. [4] demonstrated that balloon kyphoplasty (BKP), for the treatment of vertebral compression fractures, provides more rapid reduction in pain,and improves function, disability, and quality of life, as compared to non-operative treatment.

Kyphoplasty demonstrates a lower complication rate, as compared to vertebroblasty, including decreased risk of cement extravasation, pulmonary embolism, infection, epidural hematoma, and systemic toxicity [5, 6]. Paralysis following BKP is rare and has been reported to be secondary to cement extravasation [7]. Herein we report two unique cases of patients that were treated with BKP and developed instability with resultant neurological decline. To the best of our knowledge, this is the first report describing the development of instability with resultant paraplegia following BKP.

## Case report

### Case 1

**Fig. 1:**
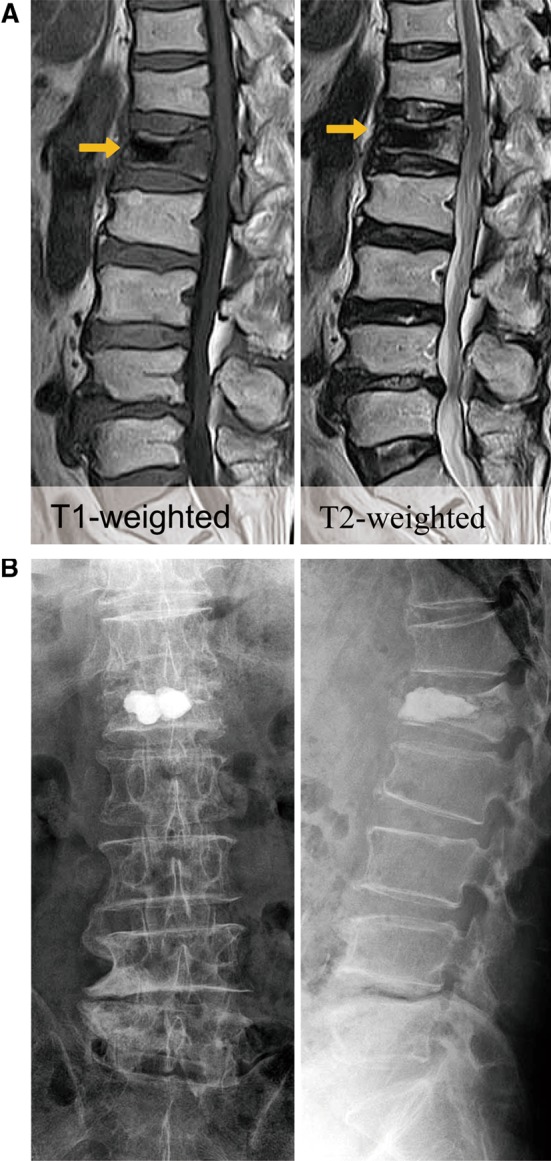
(Case 1) **a** Sagittal T1- and T2-weighted images showing collapsed vertebral body with formation of a cavitary lesion at the L1 level. **b** Radiograph of the spine after BKP reveals effective filling of cystic fracture cavity

**Fig. 2:**
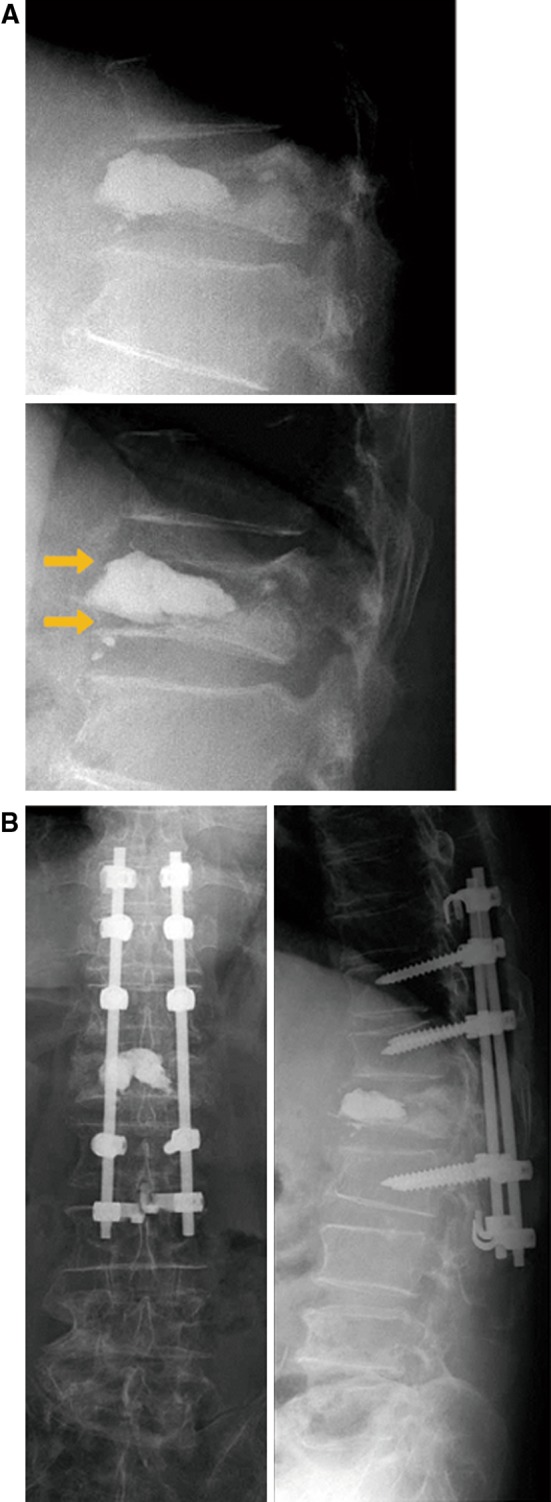
(Case 1) **a** Lateral spine flexion–extension radiographs demonstrated instability, and PMMA did not adhere to the endplate of the treated vertebrae. **b** Surgical intervention was performed by pedicle screw and hook fixation from Th10 to L2

A 72-year-old man experienced persistent severe back pain after falling from a cultivator 3 months prior to presentation. He demonstrated a normal neurological exam but was unable to ambulate secondary to pain. Magnetic resonance imaging (MRI) demonstrated a collapsed L1 vertebrae, with T1 and T2-weighted sequences showing edema and an intervertebral cleft (IVC) in the central area of the fractured vertebral body (Fig. 1a). Based on the AO classification, he was having an incomplete osteoporotic burst fracture (AO type A3.1). BKP was performed with injection of about 4.0-mL polymethyl-methacrylate (PMMA) (Fig. 1b). Postoperatively, his back pain significantly improved. At the time of hospital discharge, 2 weeks after the first operation, he was able to walk without a cane. However, he developed progressive bilateral lower-extremity weakness about 1 month after BKP. Exacerbation of back pain was not observed. Neurological examination revealed weakness in both thighs and legs (MMT 2/3), numbness on the anterior thighs, and symmetrical hyporeflexia (modified Frankel classification C1). MRI of the spine did not reveal canal stenosis but radiograph demonstrated the cement was still contained within the vertebral body. However, lateral spine flexion–extension radiographs demonstrated instability, with discontinuity between the PMMA and the endplate of the treated vertebrae (Fig. 2a). Surgical intervention was performed by instrumented fusion from T10 to L2 without decompression (Fig. 2b). Three days postoperatively, motor exam improved to grade four bilaterally with resolution of the paresthesia. Four weeks postoperatively, the patient was able to walk with a T-cane (modified Frankel classification D2).

### Case 2

**Fig. 3:**
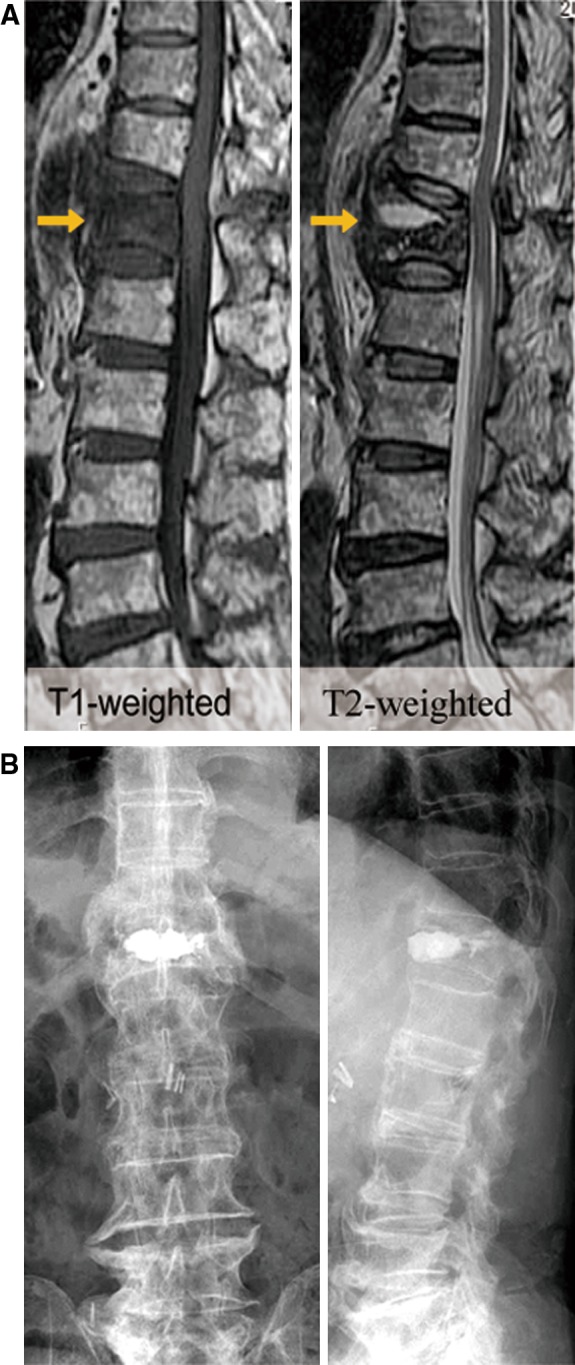
(Case 2) **a** Sagittal T1-andT2-weighted images showing collapsed vertebral body with formation of a cavitary lesion at the L1 level. **b** Radiograph of the spine after BKP reveals effective filling of cystic fracture cavity

**Fig. 4:**
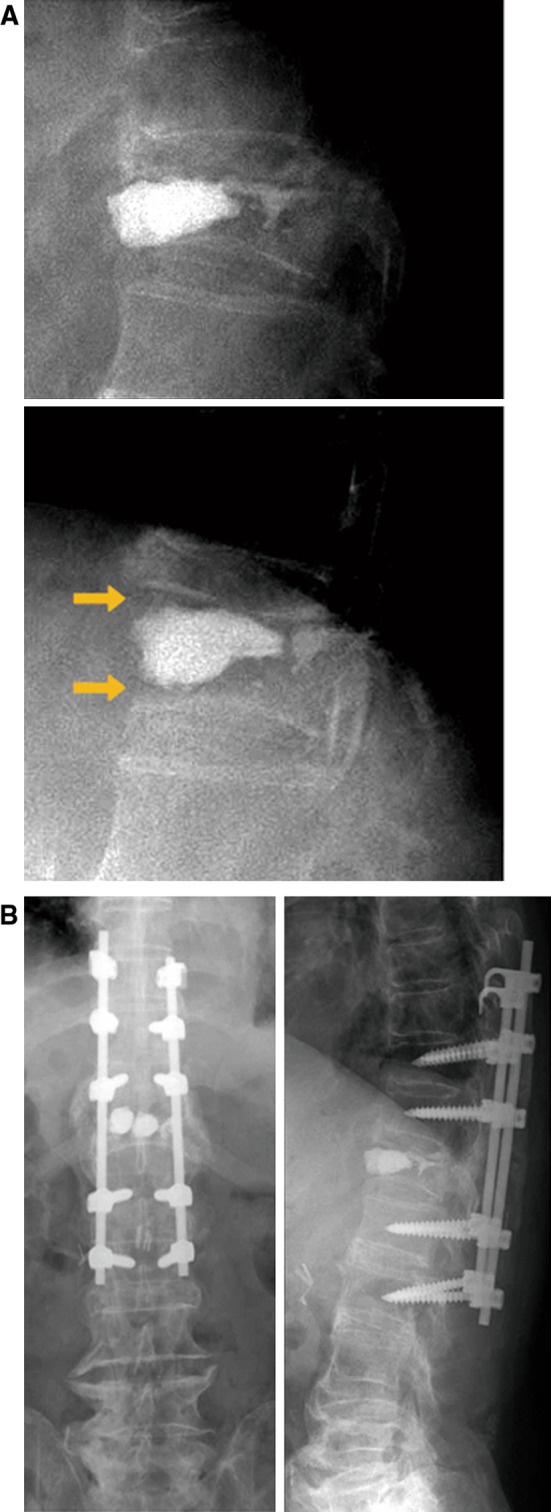
(Case 2) **a** Lateral spine flexion–extension radiographs demonstrated instability, and PMMA did not adhere to the endplate of the treated vertebrae. **b** Surgical intervention was performed by pedicle screw and hook fixation from Th10 to L2

A 79-year-old man experienced persistent severe back pain after falling from the rear deck of a truck 2 months prior to presentation. The findings on neurological examination were within normal limits. MRI of the spine revealed collapse of the vertebral body with the formation of a cavitary lesion at the T12 level. A high signal on T2-weighted MRI revealed a fluid-filled intravertebral cleft, he was having an incomplete osteoporotic burst fractures (AO type A1.3). Because of his intractable back pain, which was not relieved by conservative treatment, BKP was performed. Approximately, 6.0-mL PMMA was injected into the vertebral body (Fig. 3). Immediately after the operation, his back pain improved and he was able to walk without a cane. However, he developed progressive bilateral lower-extremity weakness at about 2 weeks after BKP. Neurological examination revealed right lower-extremity weakness (MMT 2/5), and symmetrical hyporeflexia (modified Frankel classification C2). Similar to case 1, MRI of the spine did not reveal canal stenosis, yet dynamic radiographs demonstrated instability with discontinuity between the PMMA and the endplate of the treated vertebrae (Fig. 3). An instrumented fusion was performed between T9 and L2 without decompression (Fig. 4). Postoperatively, the patient demonstrated gradual improvement in motor strength to a grade of 5/5 by 1 month (modified Frankel classification D3).

## Discussion

Vertebral compression fractures are becoming more prevalent as our society ages. Managing symptomatic osteoporotic compression fractures that fail conservative care with balloon kyphoplasty has gained in popularity. The advantages of kyphoplasty, as compared to vertebroplasty, include improved restoration of sagittal balance and decreased risk of cement extravasation secondary to the low injection pressure and increased viscosity [9]. The reported rate of cement leakage (symptomatic 0.2 %), adjacent level fracture and pulmonary embolism is 9 %, 13.6 %, 0.1 % of cases, respectively. Few case reports describe neurological complications following BKP. Previous reports of neurological complications are secondary to cement extravasation into the spinal canal [10].

The two patients described within this report had normal neurological exams during the initial perioperative period and no evidence of cement extravasation on imaging studies. Neurological decline occurred at >2 weeks postoperatively. Lavelle et al. [11] reported a 10 % incidence of recurrent fracture after kyphoplasty in the treated vertebrae typically occurring within the first 90 days postoperatively. Young-Yul Kim and colleagues suggest that inadequate filling of the intervertebral cleft, whereby the PMMA does not contact the endplates, is a risk factor for same level fracture. The two patients described in this report had an intervertebral cleft preoperatively; however, on initial radiographs it was felt that the PMMA had interdigitation with the endplates to provide the necessary load-bearing. However, in BKP, the low-pressure injection of PMMA has far less interdigitation with the surrounding bone [12, 13]. The other disadvantage of BKP is that balloon expansion and cement are usually placed in the anterior aspect of the vertebral body and thus the posterior vertebral body often lacks necessary cement and reinforcement. There was no canal stenosis observed on CT and MRI in the patients described above. However, dynamic radiographs demonstrated instability with discontinuity between the PMMA and endplate of the treated vertebrae. It was felt that the neurological deficit was secondary to dynamic stenosis and therefore the patient underwent instrumented stabilization without decompression and had complete neurological recovery. In essence there was a fracture between the endplate and the PMMA with surrounding spondylosis. Biomechanically, this created a two column fracture through the level that was treated with BKP and resultant instability. The indication for treatment of BKP is one of the most important unsolved issue case. In this report we describe one patient with an incomplete osteoporotic burst fracture (AO type A3.1) and another patient with an AO type A1.3 fracture. Walter et al. evaluated the frequency of complication with regard to the fracture type, and suggested that BKP can be considered as a safe procedure, even in the treatment of painful osteoporotic vertebral fractures of AO type A3.1 [14]. Our current case report demonstrates a cause of delayed neurological decline in those that undergoing BKP. Postoperative instability through the operated level should be considered in those who do not have evidence of a compressive lesion on MRI or CT-myelography, indicating the importance of obtaining dynamic radiographs in this group of patients.
